# The prognostic impact of tet oncogene family member 2 mutations in patients with acute myeloid leukemia: a systematic-review and meta-analysis

**DOI:** 10.1186/s12885-019-5602-8

**Published:** 2019-04-25

**Authors:** Ruiqi Wang, Xiaoning Gao, Li Yu

**Affiliations:** 1 Department of Hematology, Chinese PLA General Hospital, Medical School of Chinese PLA, 28 Fuxing Road, Beijing, 100853 China; 2 Medicine School, Nankai University, 94 Weijin Road, Tianjin, 300071 China; 3 Department of Hematology-Oncology, Carson International Cancer Center, Shenzhen University General Hospital, Shenzhen University Health Science Center, 1098 Xueyuan Avenue, Shenzhen, 518060 China

**Keywords:** *TET2* mutation, Acute myeloid leukemia, Prognosis

## Abstract

**Background:**

The impact of Tet oncogene family member 2 (*TET2*) mutations on the prognosis of acute myeloid leukemia (AML) is still controversial. A meta analysis is needed in order to assess the prognostic significance of *TET2* mutation in AML.

**Methods:**

Five databases including PubMed, Cochrane, EMBase, China National Knowledge Internet (CNKI) and Wanfang database were retrieved to search studies that investigated the correlation between *TET2* mutations and outcomes of AML patients. Pooled hazard ratios (HRs) and odds ratios (ORs) were used to assess the effects of *TET2* mutations.

**Results:**

Sixteen studies were included. *TET2* mutation was an unfavorable prognostic factor for overall survival (OS: HR = 1.386; *P* < 0.001) and event-free survival (EFS: HR = 1.594; *P* = 0.002) in patients with AML. For patients under 65 years of age, *TET2* mutation predicted an inferior OS (HR = 1.310, *P* = 0.051) and EFS (HR = 1.429, *P* = 0.027). For patients with intermediate-risk cytogenetics (IR-AML), mutant *TET2* had a significant association with adverse OS (HR = 0.474; *P* < 0.001). For patients with normal cytogenetics (CN-AML), mutant *TET2* also conferred adverse OS (HR = 1.425; *P* < 0.001) and EFS (HR = 1.450, *P* < 0.001). Further, among patients with CN-AML, mutant *TET2* was associated with inferior OS (HR = 2.034, *P* < 0.001) and EFS (HR = 2.140, *P* < 0.001) in the ELN favorable-risk subgroup and an inferior EFS (HR = 1.487; *P* < 0.001) in the ELN intermediate-Isubgroup. With respect to treatment outcome, *TET2* mutation predicted a significantly lower rate of complete remission (CR) in cases with ELN favorable-risk cytogenetics (OR = 0.460, *P* = 0.011).

**Conclusions:**

*TET2* mutation had adverse impacts on survival and treatment response in AML patients and will contribute to risk-stratification, prognosis prediction and therapy guidance.

**Electronic supplementary material:**

The online version of this article (10.1186/s12885-019-5602-8) contains supplementary material, which is available to authorized users.

## Background

Acute myeloid leukemia (AML) is the most common leukemia for its highest incidence in both new cases and deaths in leukemia. But with exploration in AML [1], people have found out several unfavorable factors that influenced the survival of patients with AML, such as cytogenetics, which have been used in risk stratification to predict the prognosis and guide therapeutic strategy [2]. Another kind of factors are genetic mutations, also used in prognostic stratification in the latest ELN risk-stratification, including mutations in genes encoding nucleophosmin 1 (*NPM1*), CCAAT/enhancer binding protein alpha (*CEBPA*), tumor protein 53 (*TP53*) and internal tandem duplication of FMS-like tyrosine kinase 3 (*FLT3*-ITD) [3].

Besides, mutations involving epigenetics also predict prognosis of AML patients despite they haven’t been published on the guideline. For instance, there are plenty of studies demonstrating that mutations in genes encoding DNA methyltransferase 3A (*DNMT3A*) and additional sex comb-like protein 1 (*ASXL1*) are poor prognostic factors on AML [4–7]. Qingyu Xu clearly upheld the adverse prognostic significance of isocitrate dehydrogenase 1 gene (*IDH1*) and the favorable prognostic significance of isocitrate dehydrogenase 2 gene (*IDH2*) respectively [8]. But tet oncogene family member 2 gene (*TET2*) still has a controversial prognostic impact on AML because studies researching *TET2* mutations are not plenty and their conclusions are not identical. From Hsiao-Wen Kao’s study [9], there was no significant difference on survival between patients with and without mutant *TET2* while Jay P. Patel developed an integrated classification system and demonstrated that *TET2* mutations were associated with shorter overall survival (OS) only among patients divided into intermediate-risk AML (IR-AML) [10]. In a meta-analysis performed by Wenjian Liu, mutant *TET2* was an unfavorable prognostic factor among not only entire AML patients but also favorable and intermediate-I subgroups based on ELN guidelines [11]. Since this meta analysis only contained 8 studies, it’s necessary to perform a updated meta analysis containing more studies to further explore the influence of *TET2* mutations on clinical outcome and survival in AML patients.

On the other hand, *TET2* has a function of converting 5-methyl-cytosine (5-mc) to 5-hydroxymethyl-cytosine (5-hmc), thus, mutant *TET2* could disorder the function of hematopoietic stem cell through epigenetic modification [12]. Drugs aiming at epigenetic mechanism have served in clinical therapy and provided a lot of benefits to patients with AML [13–16]. If the prognostic significance of *TET2* mutations can be clearly evaluated, it will be beneficial to direct individual therapeutic strategies.

## Methods

### Inclusion and exclusion criteria

Entitled studies should content following requirements: (i) constrained to human studies demonstrating the prognostic influence of mutant *TET2* on adult patients with AML. (ii) contained data in the aspect of survival and treatment outcome, (iii) published in English. Exclusion criteria were as followings: (i) focusing on pediatric AML, (ii) data which were not available or sufficient, (iii) study cohort overlapped, (iv) reviews and meta-analysis.

### Literature review

We implemented a literature search on electronic databases of PubMed, Cochrane, Embase together with Chinese databases comprising of China National Knowledge Internet and WanFang database, without limitations in publication date and regions. Terms for searching included “AML” “acute myeloid leukemia” “*TET2*” “tet oncogene family member 2”. References of articles we searched were also used in literature search.

Titles and abstracts of initially selected studies were reviewed depending on inclusion and exclusion criteria. Once contradictions occurred, they would be solved by discussion. After articles meeting demands were chosen, their full-texts would be scanned to distinguish appropriate studies and quality assessment according to Newcastle-Ottawa Scale would be used in the final procedure to determine the studies that could be included in our meta-analysis.

### Data extraction

Relevant information was extracted from ultimately determined studies and summarized. Basic extracted information included authors of studies, publication year, publication journal, region, sample size of cases, frequency of mutant *TET2*, median age of study cohort, detecting method, cohort type and therapy regimen, while clinical information contained sex ratio, laboratory results, French-American-British classification, cytogenetic risk classification, incidences of normal karyotype and complex karyotype and incidences of common genetic mutations (*NPM1*, *FLT3*-ITD, *DNMT3A*, *IDH*, *CEBPA*, *ASXL1*). Survival-related data were also extracted, including odds ratio (OR) for complete remission (CR) rates (or the cases achieving CR in patients with and without mutant *TET2*) and the hazard ratio (HR) for OS, event-free survival (EFS). Data from multivariate analyses were extracted with priority, but data from univariate analyses or calculated from Kaplan-Meier survival curves were also used in our meta-analysis when multivariate results were not available [17]. With respect to extraction of survival curves, we applied the software Engauge Digitizer 4.1 in this part. After inputting the pictures of survival curves and outputting the data of specific spots on curves, we used Excel to calculate HR and relevant data.

### Statistical analysis

We used STATA 12.0 statistical software to analyze the data extracted. *TET2* mutation was identified as relating to an unfavorable treatment outcome or survival when pooled ORs below or HRs over 1.000, which became statistically significant if 95% confidence interval (CI) did not cover 1.000 or a *P* value less than 0.05.

As for heterogeneity, Q test was used to measure it. I-square(I^2^) < 30%, 30–50%, 50–75, and > 75% meant low, moderate, substantial and considerable heterogeneity relatively [18]. Additionally, *P* < 0.10 represented a significant heterogeneity [19]. Random effect model was used for a significant heterogeneity observed, while fixed effect model was for studies without or with slight heterogeneity. Sensitivity test was used to determine the source of heterogeneity and evaluate the stability of results. Subgroup analyses were performed to evaluate the results in aspects of publication year, data type, region, study cohort, median age of patients as well as detecting method. Egger and Begg tests were used to assess publication bias, a *P* value less than 0.05 representing existence of publication bias.

## Results

### Selection procedure

**Fig. 1:**
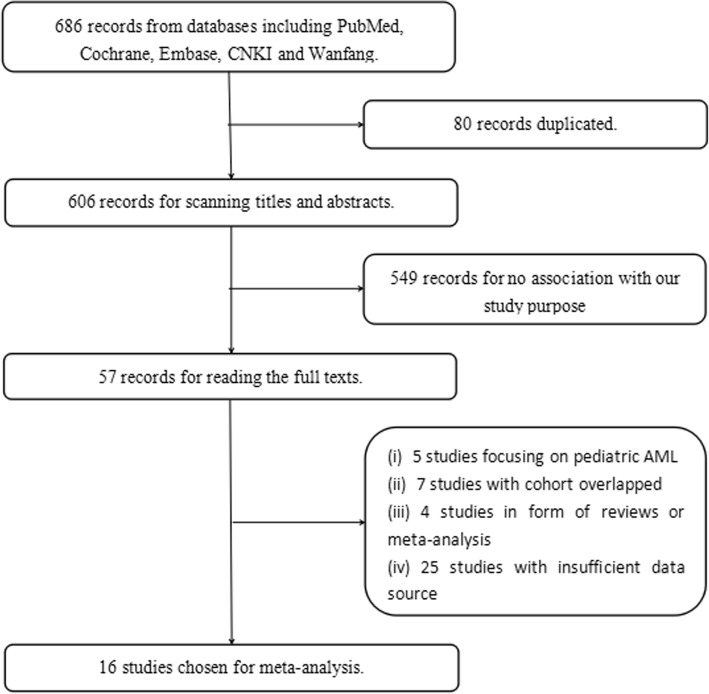
Flow chart of the study selection. CNKI, China National Knowledge Internet; AML, acute myeloid leukemia

At first, 686 studies were obtained from 5 databases, and we excluded 80 records because of duplication. Then we scanned the titles and abstracts of the remaining articles and removed 549 articles for no association with our study purpose. We read the full texts of the rest articles next and removed 41 studies due to the following reasons: 5 studies for focusing on pediatric AML, 7 studies for overlapped cohorts, 4 studies for reviews and meta-analysis, and 25 studies with incomplete data. Ultimately, there were 16 studies selected for our study. The selection procedure was illustrated in Fig. 1.

### Study characteristics

Characteristics of 16 studies with a total of 4378 cases were shown in Table 1: 4 of them from America, 6 from Europe, 5 from Asia, and one involving multiple regions [9, 10, 20–33]. *TET2* mutation had a frequency varying from 6.05 to 27.36% in AML patients and from 6.05 to 36.23% in patients with normal cytogenetics (CN-AML). Mutational frequency refers to the proportion of patients harboring *TET2* mutations.

**Table 1:** Characteristics of studies selected in the meta-analysis

Study	Year	NOS	Journal	Region	Total number	TET2m	Median age	Detecting method	Data type	Therapy regimen	Cohort type
Wahab	2009	7	Blood	America	91	11	65	Unknown	Calculated from K-M curves	Unknown	AML
Nibourel	2010	6	Blood	France	111	19	43	Direct sequencing	Calculated from K-M curves	Anthracycline-cytosine arabinoside induction treatment followed by HDAC consolidation or allo-HSCT	AML
Metzeler	2011	9	Journal of Clinical Oncology	America	418	95	> 60	Direct sequencing	Calculated from K-M curves	Standard intensive therapy	CN-AML
Kosmider	2011	7	Haematologica	France	247	49	66	Direct sequencing	Unitivariate or calculated from K-M curves	Intensive chemotherapy with anthracycline-cytarabine	s-AML
Chou	2011	7	Blood	China	486	64	51.5	Unknown	Multivariate and calculated from K-M curves	Standard intensive therapy or palliativecare or low-dose chemotherapy	AML
Patel	2012	8	The New England Journal of Medicine	America	391	33	< 60	Direct sequencing	Calculated from K-M curves	Induction therapy with high or standard dose of DNR	AML
Weissmann	2012	7	Leukemia	Germany	318	87	66.4	Next-generation sequencing	Calculated from K-M curves	Unknown	AML
Gaidzik	2012	8	Journal of Clinical Oncology	Germany	783	60	< 60	Direct sequencing	Calculated from K-M curves	Double induction therapy	AML
Renneville	2014	6	Oncotarget	France	139	19	62	Direct sequencing	Univariate	Standard front-line chemotherapy with or without low-dose gemtuzumab ozogamicin	CN-AML
Damm	2014	8	Genes Chromosomes and Cancer	France and Germany	215	13	< 60	Direct sequencing	multivariate	Intensive double induction and consolidation therapy	CN-AML
Tian	2014	7	International Journal of Hematology	Asia	373	60	45	Direct sequencing	Calculated from K-M curves	Standard induction therapy followed by consolidation of HDAC or allo-HSCT	CN-AML
S.Ohgami	2015	7	Modern Pathology	America	93	6	55	Next-generation sequencing	Muitivariate	Standard induction therapy with cytarabine and danorubicin or idarubicin	AML
Ahn	2015	9	Haematologica	Multiple region	407	54	52	Direct sequencing	Muitivariate	Standard induction therapy	CN-AML
Kao	2015	8	Oncotarget	China	98	18	55	Direct sequencing	Calculated from K-M curves	Standard intensive therapy with daunomycin and cytarabine	AML with MLL-PTD
Cher	2016	8	Blood Cancer Journal	China	96	8	41	Next-generation sequencing	Multivariate and univariate	Induction chemotherapy comprising cytarabine and daunorubicin with consolidation therapy comprising HDAC or allo-HSCT	CBF-AML
Lin	2016	9	Cancer Medicine	China	112	12	42.6	Next-generation sequencing	Multivariate	Standard therapy with or without allo-HSCT	AML

**Abbreviations:** *NOS* the Newcastle-Ottawa-Scale, *TET2,* tet oncogene family member 2, *m* mutation, *HDAC* high-dose cytarabine, *allo-HSCT* allo hematopoietic stem cell transplantation, *CN-AML* cytogenetically normal acute myeloid leukemia, *s-AML* secondary acute myeloid leukemia, *MLL-PTD* partial tandem duplication of *mixed-lineage leukemia* gene, *CBF-AML* core-binding factor acute myeloid leukemia

Eleven out of 16 studies revealed that *TET2* mutations were distributed throughout the whole coding exon detected with no specific hot spots (Additional file 2: Table S1) [9, 22–30, 32]. *TET2* mutation was significantly associated with older age in 7 studies [9, 21, 24, 25, 27, 30, 33], higher white blood cell (WBC) count in 5 studies [21, 24, 25, 27, 30], higher hemoglobin level in 2 studies [30, 33] and with lower platelet count in 4 studies (Additional file 3: Table S2) [25, 27, 28, 30]. Besides, one study showed that *TET2* mutations were rarer in M3 whereas another showed *TET2* mutation was more frequent in M4 with respect to FAB classification [25, 28]. The others didn’t show any relationship between mutant *TET2* and FAB subtypes (Additional file 3: Table S3). In addition, *TET2* mutation was more frequent in AML patients with normal karyotype according to two studies [25, 30], and there was only one study showing the close association between mutant *TET2* and intermediate-risk cytogenetics (Additional file 3: Table S3) [25]. But after a meta-analysis, we found a significantly close relationship between *TET2* mutations and intermediate-risk cytogenetics with an OR of 1.765(95%CI: 1.212–2.569; *P* = 0.003; I^2^ = 39.7%, *P* = 0.159), and *TET2* mutations were more infrequent in favorable-risk cytogenetics(OR = 0.547, 95%CI: 0.325–0.920; *P* = 0.023; I^2^ = 11.2%, *P* = 0.337). As for gene-gene association, a significantly higher frequency of *NPM1* mutation was existed in patients with *TET2* mutations compared with those with wild type *TET2* according to 5 studies [21, 25, 28, 29, 31]. The same strong relationship was also noted between mutant *ASXL1* and mutant *TET2* in 3 studies [10, 20, 25]. On the contrary, *IDH* mutation was mutually excluded with *TET2* mutation, which was observed in 7 studies (Additional file 3: Table S5) [9, 10, 24–26, 28, 33], and the pooled OR was 0.123(95%CI: 0.071–0.212; *P* < 0.001). Then we conducted meta analyses and explored the exact close association of *TET2* mutations with *NPM1*(OR = 1.735, *P* < 0.001), *DNMT3A*(OR = 2.361, *P* = 0.045) and *ASXL1* mutations(OR = 2.743, *P* = 0.009).

### Prognosis of *TET2* mutation in AML

**Fig. 2:**
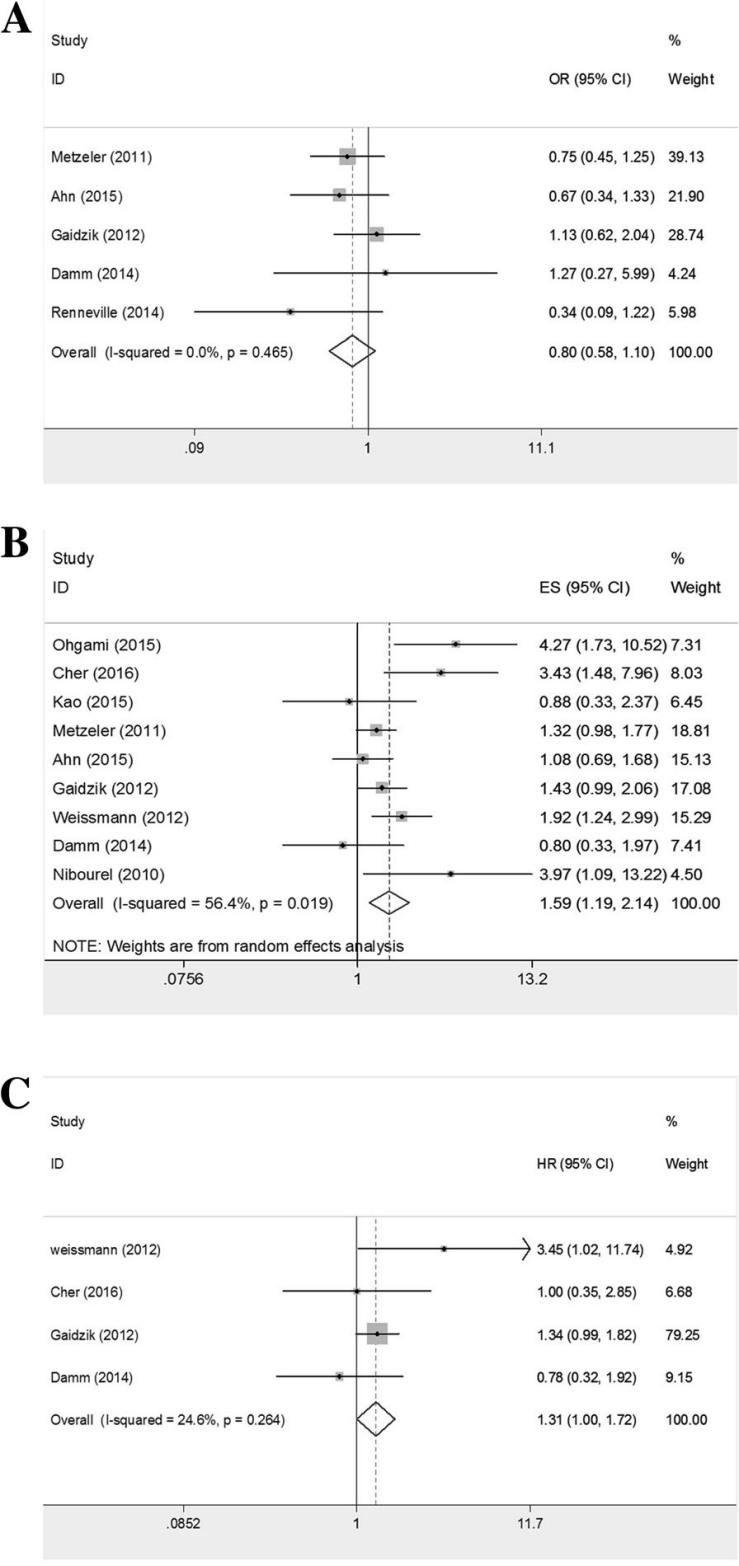
Prognosis of AML patients with *TET2* mutation. **a** pooled ORs and 95%CI for CR rate(OR 0.802; *P* = 0.176). **b** pooled HRs and 95%CI for EFS(HR 1.594; *P* = 0.002). **c** pooled HRs and 95%CI for OS in patients under 65 years of age(HR 1.310; *P* = 0.051)

With respect to response to therapy in AML patients, the combined ORs of *TET2* mutation for CR rate were 0.802 (95%CI: 0.583–1.103; *P* = 0.176; heterogeneity: I^2^ = 0.0%, *P* = 0.465; Fig. 2a). Fifteen studies were included to calculate pooled HRs for OS in AML patients, resulting in the result of 1.480 (95%CI: 1.241–1.766; *P* < 0.001; heterogeneity: I^2^ = 34.3%, *P* = 0.094). With *P* < 0.1 in heterogeneity test, we conducted a sensitivity test and found recombined HRs for OS were 1.386 (95%CI: 1.217–1.577; *P* < 0.001; heterogeneity: I^2^ = 19.7%, *P* = 0.239) when Lin’s study were excluded (Additional file 1: Figure S1a-1b). Considering EFS in AML patients, we combined 9 studies and pooled HRs were 1.594 (95%CI: 1.187–2.141; *P* = 0.002; heterogeneity: I^2^ = 56.4%, *P* = 0.019; Fig. 2b). We conducted a sensitivity test owing to the significant heterogeneity and that result wasn’t altered by omitting any one of the studies. We therefore conducted subgroup analyses to evaluate the result (Table 2). Furthermore, we also explored the impact of mutant *TET2* on OS in AML patients under 65 years of age, figuring out a pooled HRs of 1.310 (95%CI: 0.999–1.718; *P* = 0.051; heterogeneity: I^2^ = 24.6%, *P* = 0.264; Fig. 2c), while the pooled HRs for EFS in the same cohort were 1.724 (95%CI: 1.007–2.954; *P* = 0.047; heterogeneity: I^2^ = 56.1%, *P* = 0.077) and became 1.429 (95%CI: 1.041–1.962, *P* = 0.027; heterogeneity: I^2^ = 37.4%, *P* = 0.202) after the study of Cher and colleagues was excluded (Additional file 1: Figure S1c-1d). Interestingly, we found that the summary HRs for OS in patients with IR-AML were 1.662 (95%CI: 1.312–2.105; *P* < 0.001; heterogeneity: I^2^ = 46.1%, *P* = 0.046), and decreased to 1.474 (95%CI: 1.252–1.734; *P* < 0.001; heterogeneity: I^2^ = 17.1%, *P* = 0.285) with the study of Patel and colleagues omitted (Additional file 1: Figure S1e-1f).

**Table 2:** Subgourp analyses of EFS on TET2 mutation

Variables		Number of studies, heterogeneity *I*^*2*^%, *p*	Pooled HRs(95% CI), *P* value	Interaction(*p*)
Year	2016	1	3.430[1.479–7.955], *P* = 0.004	0.096
	2015	3(74.8), P = 0.019	1.320[0.911–1.913], *P* = 0.142	
	2014	1	0.800[0.327–1.955], *P* = 0.624	
	2012	2(1.7), *P* = 0.313	1.613[1.217–2.138], *P* = 0.001	
	2011	1	1.320[0.982–1.774], *P* = 0.066	
	2010	1	3.970[1.140–13.826], *P* = 0.030	
Data type	Multivariate	3(76.4), *P* = 0.014	1.284[0.891–1.850], *P* = 0.181	0.110
	Calculated from K-M curves	5(26.2), *P* = 0.247	1.473[1.209–1.794], *P* < 0.001	
	univariate	1	3.430[1.479–7.955], *P* = 0.004	
Region	America	2(83.0), *P* = 0.015	1.479[1.117–1.959], *P* = 0.006	0.413
	Europe	4(44.1), *P* = 0.147	1.580[1.215–2.055], *P* = 0.001	
	Asia	2(76.4), *P* = 0.040	1.934[1.020–3.667], *P* = 0.043	
	other	1	1.076[0.688–1.682], *P* = 0.748	
Cohort	AML	2(79.4), *P* = 0.028	1.669[1.189–2.344], *P* = 0.003	0.444
	CN-AML	5(47.5), *P* = 0.106	1.377[1.120–1.692], *P* = 0.002	
	Others	2(76.4), P = 0.040	1.934[1.020–3.667], *P* = 0.043	
Detection methods	Direct sequencing	6(13.1), *P* = 0.331	1.283[1.058–1.556], *P* = 0.011	0.002
	Next-generation sequencing	3(38.3), *P* = 0.198	2.418[1.691–3.459], *P* < 0.001	

**Abbreviations:** *HRs* hazard ratios, *CN-AML* cytogenetically normal acute myeloid leukemia

### Prognosis of *TET2* mutation in CN-AML

**Fig. 3:**
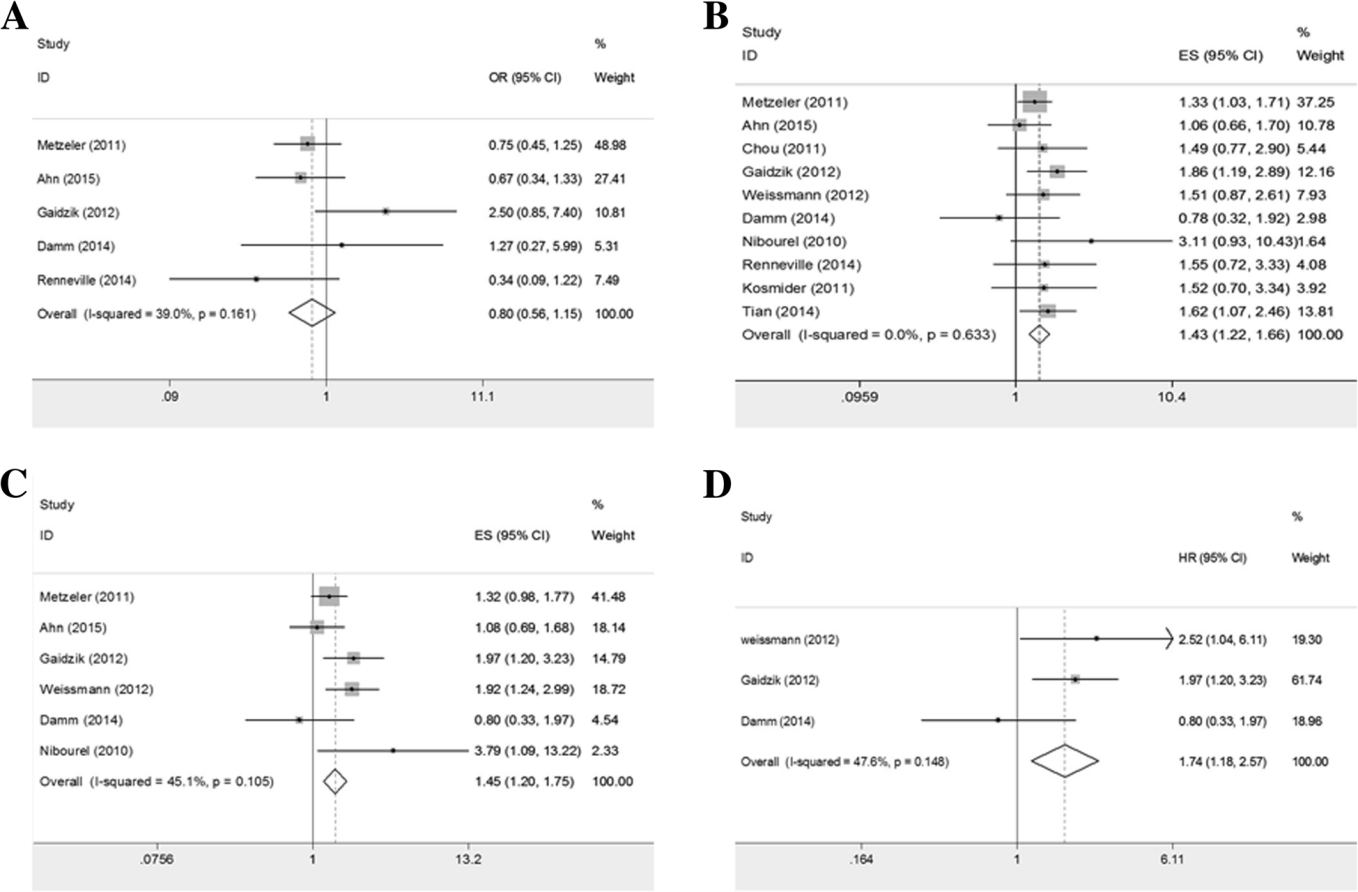
Prognosis of CN-AML patients with *TET2* mutation. **a** pooled ORs and 95%CI for CR rate(OR 0.803; *P* = 0.228). **b** pooled HRs and 95%CI for OS(HR 1.425; *P* < 0.001). **c** pooled HRs and 95%CI for EFS(HR 1.450; *P* < 0.001). **d** pooled HRs and 95%CI for EFS in patients under 65 years of age(HR 1.741; *P* = 0.005)

Among patients with CN-AML, the pooled ORs of *TET2* mutation for CR rate were 0.803 (95%CI: 0.562–1.147; *P* = 0.228; heterogeneity: I^2^ = 39.0%, *P* = 0.161; Fig. 3a). Despite the insignificance of ORs for CR rate, integrated HRs for OS were 1.425 (95%CI: 1.221–1.664; *P* < 0.001; heterogeneity: I^2^ = 0.0%, *P* = 0.633; Fig. 3b), while the pooled HRs for EFS were 1.450 (95%CI: 1.199–1.754; *P* = 0.001; heterogeneity: I^2^ = 45.1%, *P* = 0.105; Fig. 3c).

Among patients with CN-AML under 65 years of age, the pooled HRs for OS were 1.630 (95%CI: 0.817–3.250; *P* = 0.166; heterogeneity: I^2^ = 54.0%, *P* = 0.114) and became 1.999 (95%CI: 1.317–3.033, *P* = 0.001; heterogeneity: I^2^ = 0.0%, *P* = 0.352)(Additional file 1: Figure S2a-2b) with Damm’s study omitted, while the combined HRs for EFS were 1.741 (95%CI: 1.180–2.569; *P* = 0.005; heterogeneity: I^2^ = 47.6%, *P* = 0.148; Fig. 3d).

### Prognosis of *TET2* mutation based on ELN risk stratification

**Fig. 4:**
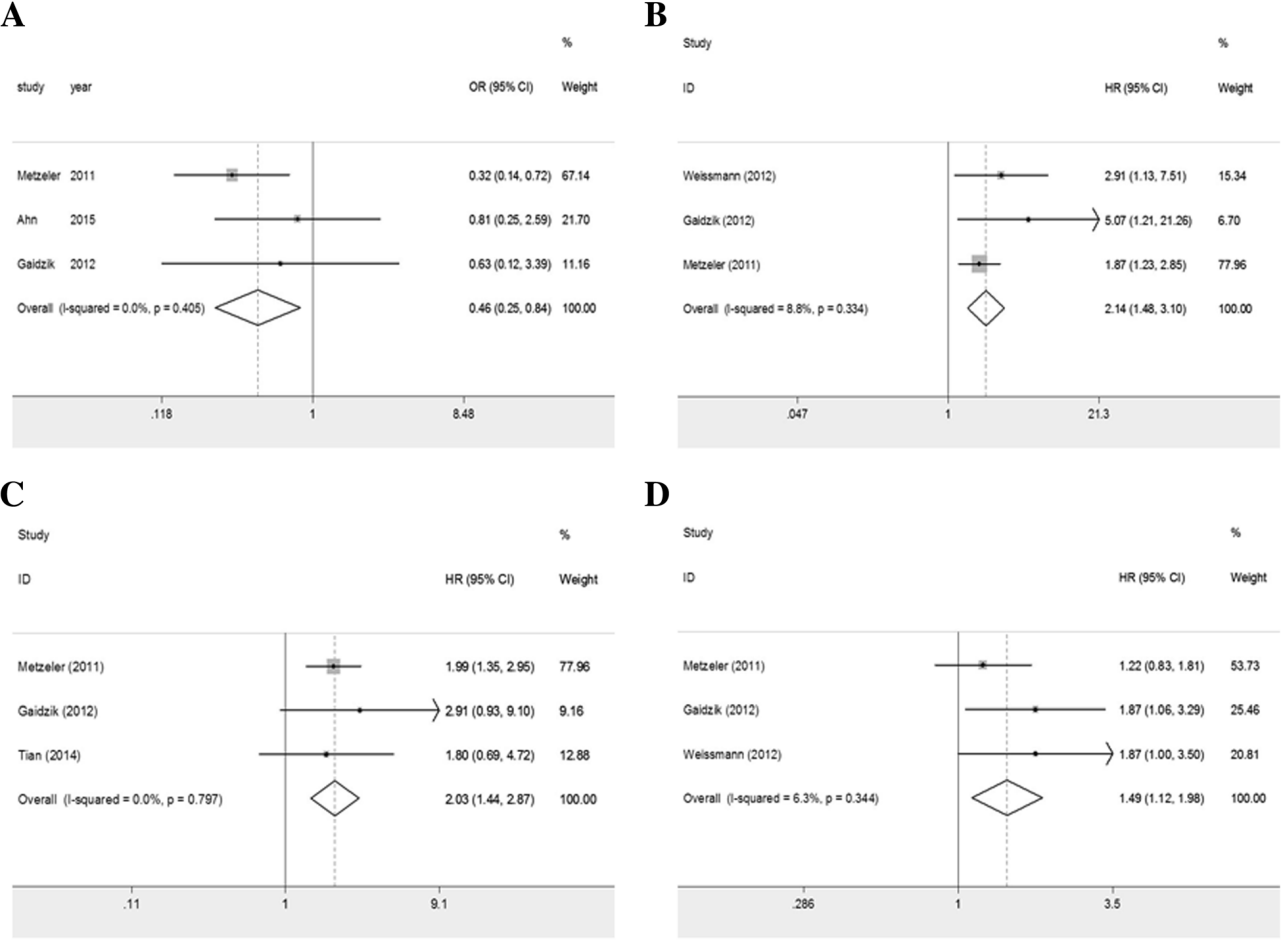
Prognosis of AML patients with *TET2* mutation according to ELN risk-stratification. **a** pooled ORs and 95%CI for the CR rate in patients with ELN favorable-risk cytogenetics(OR 0.460; *P* = 0.011). **b** pooled HRs for OS in patients with ELN favorable-risk cytogenetics(HR 2.034; *P* < 0.001). **c** pooled HRs and 95%CI for EFS in patients with ELN favorable-risk cytogenetics(HR 2.140; *P* < 0.001). **d** pooled HRs and 95%CI for EFS in patients with ELN intermediate-risk cytogenetics

We evaluated the prognosis of *TET2* mutation based on ELN risk stratification. Among patients in ELN favorable-risk (CN-AML patients carrying *NPM1*mutwithout *FLT3*-ITD and/or *CEBPA*double mut), the combined ORs for CR rate were 0.460 (95%CI: 0.252–0.840; *P* = 0.011; heterogeneity: I^2^ = 0.0%, *P* = 0.405; Fig. 4a). What’s more, the pooled HRs for EFS were 2.140 (95%CI: 1.476–3.101; *P* < 0.001; heterogeneity: I^2^ = 8.8%, *P* = 0.334; Fig. 4b). The pooled HRs for OS were 2.034 (95%CI: 1.440–2.872; *P* < 0.001; heterogeneity: I^2^ = 0.0%, *P* = 0.797; Fig. 4c). The results above showed an unfavorable prognostic impact of *TET2* mutation in patients of this group. As for patients in ELN intermediate-Irisk group (all remaining patients with CN-AML), the pooled ORs for CR rate were 1.158 (95%CI: 0.408–3.280; *P* = 0.783; heterogeneity: I^2^ = 70.5%, *P* = 0.034). Hence, we performed a sensitivity test as well although the number of studies included was only three. After excluding the study of Gaidzik and colleagues, the recombined ORs for CR rate decreased to 0.809 (95%CI: 0.465–1.409; *P* = 0.454; heterogeneity: I^2^ = 45.7%, *P* = 0.175) (Additional file 1: Figure S3a-3b). Therefore, this study made up a part of the significant heterogeneity and has to be discussed further. In addition, the pooled HRs for EFS were 1.487 (95%CI: 1.117–1.978; *P* = 0.007; heterogeneity: I^2^ = 6.3%, *P* = 0.344; Fig. 4d).

### Sensitivity analysis

Among patients with AML, the study of Lin and colleagues made up a part of heterogeneity due to its outlier of HR for OS according to the sensitivity test, but it shouldn’t be excluded for the following reasons: At first, the sample size of cases from the entire cohort was adequate as well as the number of cases with *TET2* mutation; Second, the maximum follow-up period of this study was around 120 months, which was long enough to diminish the bias; Next, the new result didn’t change too much with this study omitted, and *TET2* mutation still conferred an unfavorable impact on OS in AML patients. Likewise, among patients with IR-AML, the sensitivity test showed that the study of Patel and colleagues was the biggest contribution of heterogeneity in pooled HRs for OS. This study showed a relatively stronger impact of *TET2* on OS compared with other studies, but actually there was no need to exclude it due to the reasons above. More importantly, this study didn’t influence pooled HRs of *TET2* mutation for the OS significantly. With regard to patients under 65 years of age, the pooled HRs for EFS decreased from 1.724 to 1.429 with a lower heterogeneity after the study of Cher and colleagues was excluded. The sample size of cases with *TET2* mutation from Cher’s study was the smallest, which might be expected to result in the heterogeneity.

In patients with CN-AML under 65 years of age, significant heterogeneity in pooled HRs for OS was from Damm’s study. Actually, this study demonstrated a totally different result opposed to other two studies confirming the unfavorable impact of *TET2* mutation in this cohort. The relatively lower percentage (6.75%) and fewer cases (11 cases) with *TET2* mutation might be the reasons for the bias.

As for patients in ELN intermediate-Irisk group, the study of Gaidzik and colleagues could account for a part of the significant heterogeneity in pooled ORs for CR rate and the combined ORs decreased to 0.809 from 1.120 with this study excluded. The cohort in Gaidzik’s study had an age range from 18 to 60 years, nevertheless the patients from the other two studies had more broad age range (Ahn: from 15 to 84 years; Metzeler: from 18 to 83 years), including patients over 60 years old. Therefore the relatively younger patient cohort could explain the heterogeneity in OR for CR rate and might be the cause of relatively higher CR rate in Gaidzik’s study. The study of Gaidzik and colleagues had a large sample size of cases with *TET2* mutation and a relatively long follow-up period. Hence, this study should be reserved and the impact on CR rate of *TET2* mutation in patients with ELN intermediate-Irisk cytogenetics remained unclear.

### Subgroup analyses

As we stated previously, we performed subgroup analyses to further assess the prognosis of *TET2* mutation on EFS in AML patients. We found that publication year, data types, regions of samples and study cohorts had no influence on OS for mutant *TET2*. In aspect of detection method, the studies using next-generation sequencing method had more significant results compared with those using direct sequencing method (Table 2). Direct sequencing couldn’t detect all the mutated spots and sometimes might miss the real *TET2* mutation, while next-generation sequencing skill could detect all the genome, more sensitive and precise than direct sequencing, so it could reduce the incident of false-negative events and exactly represent the adverse impact of *TET2* mutation.

### Publication bias

We performed Egger tests and Begg tests to assess publication bias in this study (Additional file 3: Table S6). There existed no publication bias in other results in both Egger test and Begg test (Additional file 1: Figure S4A-S4D).

### Comparing results from fixed effect model with those from random effect model

With respect to OS and EFS for mutant *TET2*, HRs and 95%CI without heterogeneity (I^2^ = 0.00%) from the fixed effect model were the same as those from the random effect model. HRs and 95%CI with heterogeneity (I^2^ > 0.00%) from the fixed effect model had slight changes compared with those from the random effect model but these changes did not influence the prognosis analyses. In addition, ORs and 95%CI for CR rate were slightly changed from the fixed effect model to the random effect model no matter whether there existed heterogeneity or not, which had no impact on prognosis analyses (Additional file 3: Table S7).

## Discussion

### Major findings

In this study, *TET2* mutations exhibited markedly unfavorable impacts on prognosis of AML patients. Patients with *TET2* mutations had reduced OS (HR: 1.386; *P* < 0.001) compared with those with wild type *TET2*. We also observed that mutant *TET2* had unfavorable impacts on OS (HR: 1.310, *P* = 0.051) and EFS (HR: 1.429, *P* = 0.027) in patients under 65 years of age. Although 95%CI for OS covered 1.000, the *P* value was slightly over 0.050 and still conferred an adverse trend for OS. In addition, *TET2* mutations could also significantly reduce the OS of patients with IR-AML (HR: 1.474, *P* < 0.001).

Likewise, the same adverse influences of *TET2* mutations could be observed in patients with CN-AML (HR for OS: 1.425, *P* < 0.001; EFS: 1.450, *P* < 0.001). Among CN-AML patients, patients under 65 years of age harboring *TET2* mutations had more reduced OS (HR: 1.999, *P* = 0.001) and EFS(HR: 1.741, *P* = 0.005) compared with those harboring wild type.

As patients with CN-AML could be subdivided into a favorable group (ELN favorable-risk group) and an unfavorable-risk group (ELN intermediate-Igroup), we then evaluated the prognostic influence of *TET2* mutation on patients from different ELN subgroups. Among ELN favorable-risk group, *TET2* mutations led to significantly decreased CR rate (OR: 0.460, *P* = 0.011) and reduced survival (HR for EFS: 2.140, *P* < 0.001; HR for OS: 2.034, *P* < 0.001), which showed the adverse impact of *TET2* mutation in this group. As for patients in ELN intermediate-Igroup, because of the significant heterogeneity, we performed a sensitivity analysis and found that recombined ORs for CR rate was 0.809 after excluding Gaidzik’s study but still didn’t reach statistically significance. We had discussed that the diverse age range might be the cause for the higher CR rate of patients from Gaidzik’s study. According to several studies, age could be an independent prognostic factor in AML, and younger patients had a better treatment outcome compared with older patients. However, this study shouldn’t be excluded due to the large sample size of cases with mutant *TET2* (23.3%) and the relatively long follow-up period in this study. Therefore, the result needs to be confirmed further. In addition, mutant *TET2* had an unfavorable impact on EFS of patients in ELN intermediate-Igroup (HR: 1.487, *P* < 0.001). Because the hot spots of *TET2* mutations were not explored in the whole exon, we were not able to evaluate the prognostic impact of the specific mutated spot of *TET2*.

The emergence of *TET2* mutations represents an early event in the progression from normal hematopoiesis to AML. Nonetheless, some patients with mutations in AML-associated genes such as *TET2* don’t develop AML. Many researchers proposed a conception of clonal hematopoiesis of indeterminate potential, or CHIP, which was linked to a very low rate of conversion to AML and was also age-related like *TET2* mutations. However, it’s possible that all clones in CHIP would generate AML if given enough time and additional hits of genetic mutations. That might account for this relationship between *TET2* mutations and elder age.

Our study explored the adverse prognostic effect of mutant *TET2* on AML patients based on aspects of patients cohort, age, and risk stratification, which can bring great benefits to prognosis evaluation and therapy strategy guidance. In Itzykson’s study, azacitidine conferred a better response rate on patients with myelodysplastic syndromes and low blast count AML, which showed *TET2* mutations’ predictive value of response to hypomethylated agents [34]. We also validated the superiority of next-generation sequencing skill compared with direct sequencing skill from the subgroup analyses of EFS in AML patients, which could predict the promising prospect of next-generation sequencing skill in gene detection due to its precision and a high sensitivity.

### Comparison with other analyses

Our study confirmed the adverse effects of mutant *TET2* on prognosis of AML patients in accordance with another meta-analysis which was operated by Liu Wenjian with 8 studies included [11]. Both studies evaluated OS and EFS of patients from the entire AML, CN-AML, ELN favorable-risk group and intermediate-Igroup. Our meta analysis included 16 studies containing 4577 cases altogether, more stable and more reliable than the previous analysis. Our research was built on a broad population involving many regions including America, Asia, Europe, which meant utilization.

Besides, we summarized the correlation of *TET2* mutations and several major clinical features involving age, laboratory parameters, FAB subtypes, cytogenetics and other genetic alterations. We noted that mutant *TET2* correlated with older age, higher WBC count, lower platelet count, intermediate-risk cytogenetics, mutations in *NPM1*, *DNMT3A* and *ASXL1*, and were mutually excluded with favorable-risk cytogenetics and *IDH* mutations.

More importantly, we explored more about prognostic influences of mutant *TET2* on survival and treatment outcomes. *TET2* mutation had negative impacts on CR rate (OR: 0.460, *P* = 0.011) and OS (HR: 2.034, *P* < 0.001) within patients in ELN favorable-risk group. Nevertheless, among patients with AML, CN-AML and those in ELN intermediate-Igroup, *TET2* mutation had no impact on CR rate. We also investigated the correlation of *TET2* mutation with OS and EFS of patients under 65 years of age, and that mutant *TET2* was linked to unfavorable OS and EFS in this kind of patients cohort with AML (HR for OS: 1.310, *P* = 0.051; EFS:1.429, *P* = 0.027) and CN-AML (HR for OS: 1.999, *P* = 0.001; EFS: 1.741, *P* = 0.005). We also found the adverse impact of mutant *TET2* on OS (HR: 1.474, *P* < 0.001) in patients with IR-AML. All the findings above were not presented in previous meta analysis.

In addition, we operated subgroup analyses to evaluate the stability of our results deeply. We found that next-generation sequencing skill could lead to a more significant HR for EFS of *TET2* mutation.

### Limitations of our study

Regardless of the fact that we made the effort to perfect our study, there still existed several limitations. To start with, we only brought studies in English and Chinese into our research, with studies in other kinds of languages omitted, which might influence the real consequences. Secondly, all the studies included were retrospective studies, which made it difficult control the selective criteria and guarantee homogeneity of chosen studies. Thirdly, although some of studies provided precious data of HRs and 95%CI in multivariate analyses, there were also some studies only providing data from univariate analyses, even some data needed extracting from Kaplan-Meier survival curves, all of which might reduce the stability of the results. Next, various studies included in our meta analysis didn’t provide the specific information about karyotype, WHO subtype, FAB subtype, genetic aberration and survival period of each patient. Therefore we couldn’t investigate the relationship between the impact of *TET2* mutations on prognosis and the factors above. Finally, we were not capable of evaluate the effect of mutant *TET2* on prognosis in patients over 65 years old due to the data deficiency, which made it difficult to compare the impact on survival of *TET2* mutation between different age groups. In addition, some forest graphs in our analysis only contained 3 individual studies, which appeared to compromise the overall accuracy. Nonetheless, we rigidly operated the accurate procedures of meta analysis and that all included studies were evaluated of high quality. What’s more, after omitting the study of high heterogeneity, the width of confidence interval reduced, which meant that the accuracy actually increased.

## Conclusions

Although there were still controversies over the clinical significance of *TET2* mutations according to different conclusions from a variety of studies, we confirmed the unfavorable impacts of *TET2* mutations on response to therapy and prognosis in AML stratified by age or cytogenetics. Research on *TET2* mutations will provide more benefits for risk stratification, prognosis judgment and therapy guideline. We believe that further study of genetic aberrances and the development of next-generation sequencing skill will promote our perception of malignant hematologic neoplasms in development mechanism and risk factors.

## Additional files

Additional file 1: **Figure S1**: Title of data: Influences of *TET2* mutation on prognosis in AML patients. Description of data: forest plots of pooled HRs and 95%CI for OS or EFS in AML patients. **Figure S2**: Title of data: Influences of *TET2* mutation on prognosis in CN-AML patients. Description of data: forest plots of pooled HRs and 95%CI for OS or EFS in CN-AML patients. **Figure S3**: Title of data: Influences of *TET2* mutation on prognosis in patients with ELN intermediate-Irisk cytogenetics. Description of data: forest plots of pooled HRs and 95%CI for OS or EFS of patients with ELN intermediate-Irisk cytogenetics. **Figure S4**: Title of data: Funnel plot for publication bias test of *TET2* mutation in EFS. (DOC 916 kb)

Additional file 2: **Table S1**. Specific mutational spots of *TET2* gene collected from 11 studies. (XLS 79 kb)

Additional file 3: **Table S2**: Title of data: Patients characteristics from the studies according to *TET2* gene status. **Table S3**: Title of data: The relationship between *TET2* mutation and FAB subtypes. **Table S4**: Title of data: The relationship between *TET2* mutation and cytogenetics. **Table S5**: Title of data: The relationship between *TET2* mutation and other genetic aberrations. **Table S6**: Title of data: The results of Egger test and Begg test to evaluate publication bias. **Table S7**: Title of data: The results comparison between the fixed effect model and the random effect model. (DOC 356 kb)

## Abbreviations

5-hmc: 5-hydroxymethyl-cytosine
5-mc: 5-methyl-cytosine
AML: Acute myeloid leukemia
CHIP: Clonal hematopoiesis of indeterminate potential
CI: Confidence interval
CN-AML: AML with normal cytogenetics
EFS: Event-free survival
HR: Hazard ratio
IR-AML: AML with intermediate-risk cytogenetics
OR: Odds ratio
OS: Overall survival
WBC: White blood cell
