# Supplementary material for: The prognostic impact of tet oncogene family member 2 mutations in patients with acute myeloid leukemia: a systematic-review and meta-analysis
Source: BMC Cancer. 2019 Apr 25;19:389. doi: 10.1186/s12885-019-5602-8 (PMC6485112; doi:10.1186/s12885-019-5602-8)

**Supplementary Figures**

**Supplementary Figure S1**. Influences of TET2 mutation on prognosis in AML patients. a)pooled HRs and 95%CI for OS in AML patients(HR 1.480; *P*<0.001). b) pooled HRs and 95%CI for OS in AML patients with the study of Lin et al. Omitted(HR 1.386; *P*<0.001). c) pooled HRs and 95%CI for EFS in patients under 65 years of age(HR 1.724; *P*=0.047). d) pooled HRs and 95%CI for EFS in patients under 65 years of age with the study of Cher et al. omitted(HR 1.429; *P*=0.027). e) pooled HRs and 95%CI for OS in patients with intermediate-risk cytogenetics(HR 1.680; *P*<0.001). f) pooled HRs and 95%CI for OS in patients with intermediate-risk cytogenetics with the study by Patel et al. omitted(HR 1.503; *P*<0.001).

**Supplementary Figure S2**. Influences of TET2 mutation on prognosis in CN-AML patients. a) pooled HRs and 95%CI for OS in patients under 65 years of age(HR 1.630; *P*=0.166). b) pooled HRs and 95%CI for OS in patients under 65 years of age with the study of Damm et al. omitted(HR 1.999; *P*=0.001).

**Supplementary Figure S3**. Influences of TET2 mutation on prognosis in patients with ELN intermediate-Ⅰrisk cytogenetics. a) pooled ORs and 95%CI for CR rate(OR 1.158; *P*=0.783). b) pooled ORs and 95%CI for CR rate with the study by Gaidzik et al. exluded(OR 0.809; *P*=0.454).

**Supplementary Figure S4**. Funnel plot for publication bias test of TET2 mutation in EFS. a) in AML patients. b) in CN-AML patients. c) in patients with ELN favorable-risk cytogenetics. d) in patients with intermediate-Ⅰrisk cytogenetics.

Supplementary Figure S1.

1. b.


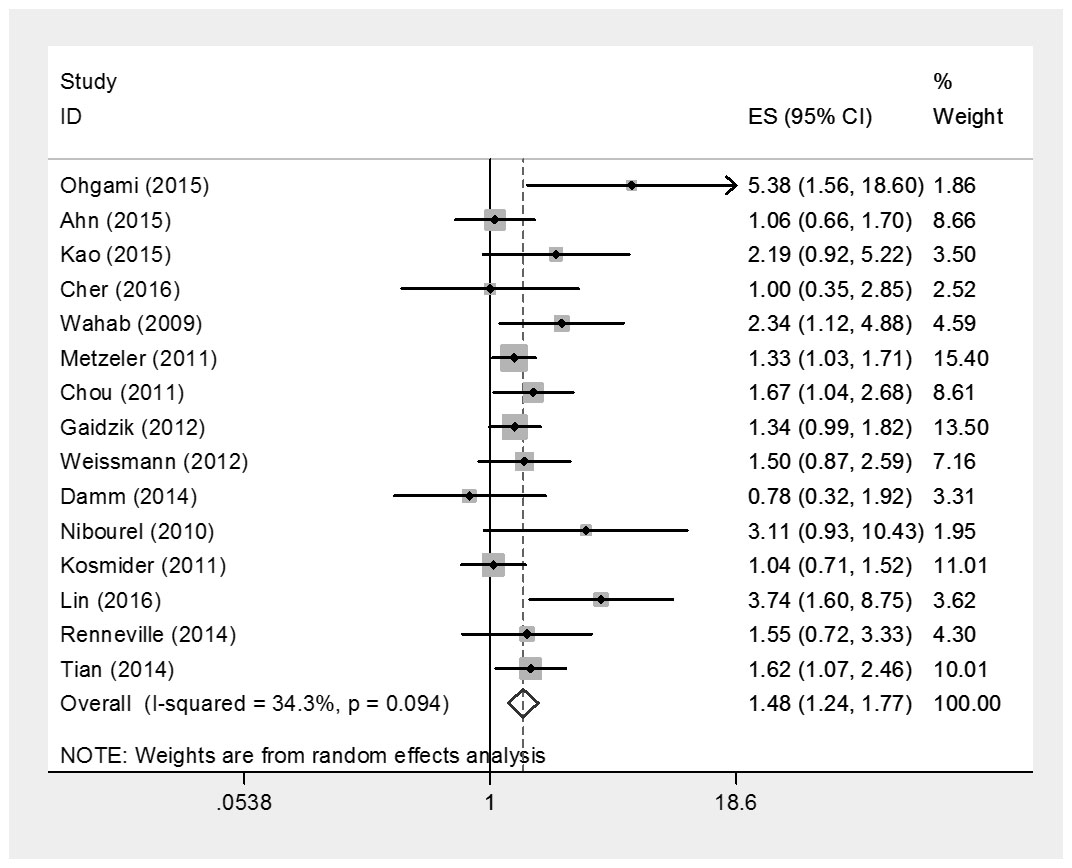

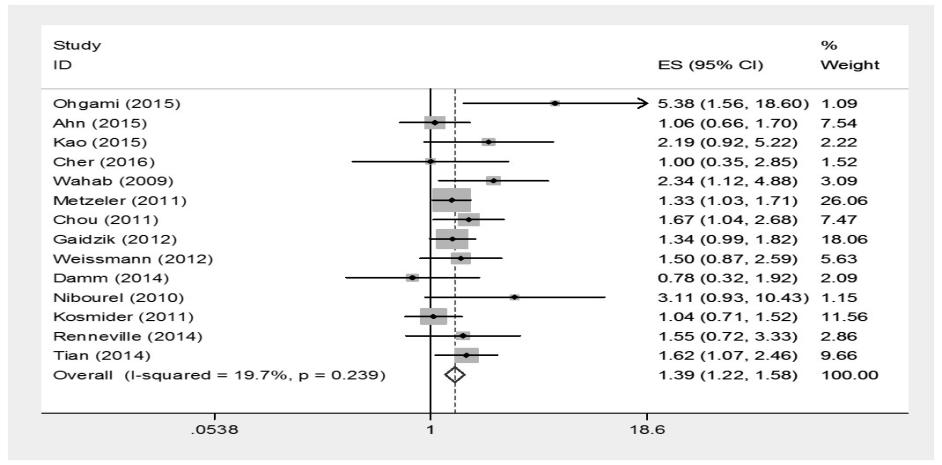


c. d.


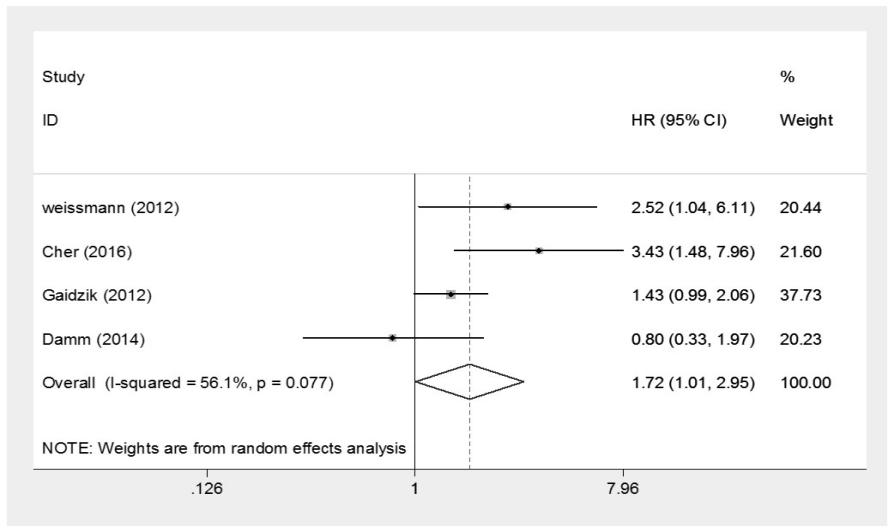

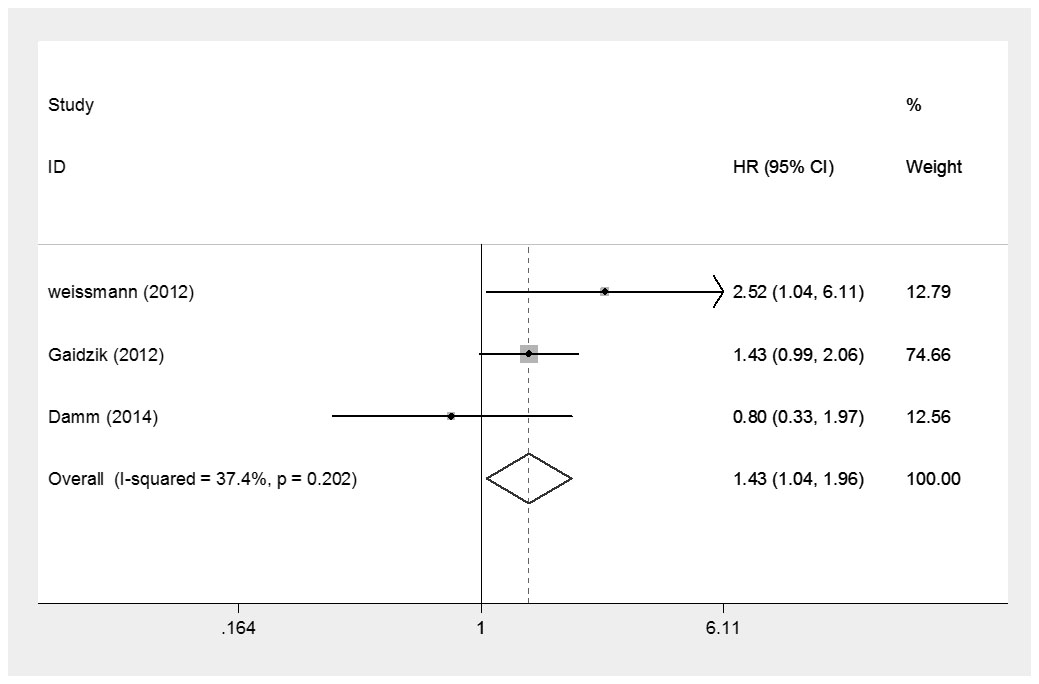


e. f.


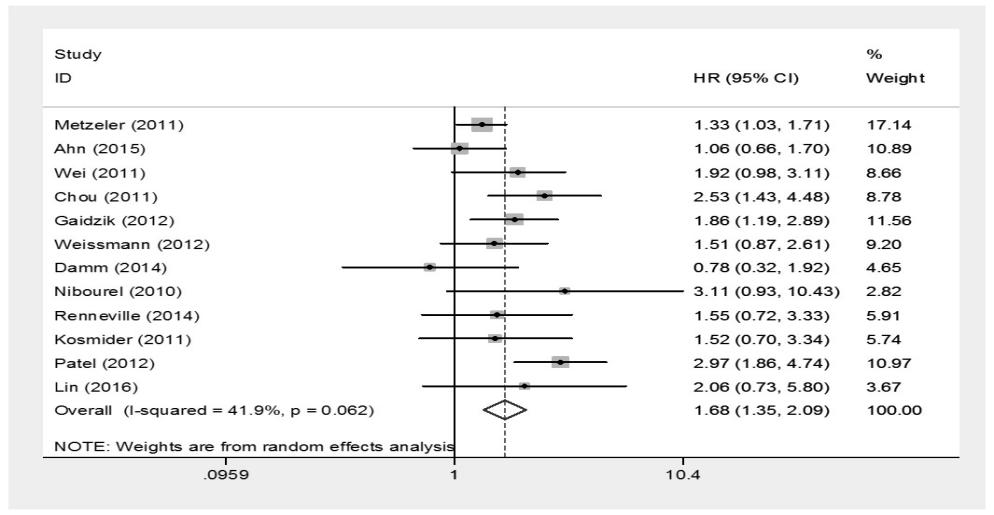

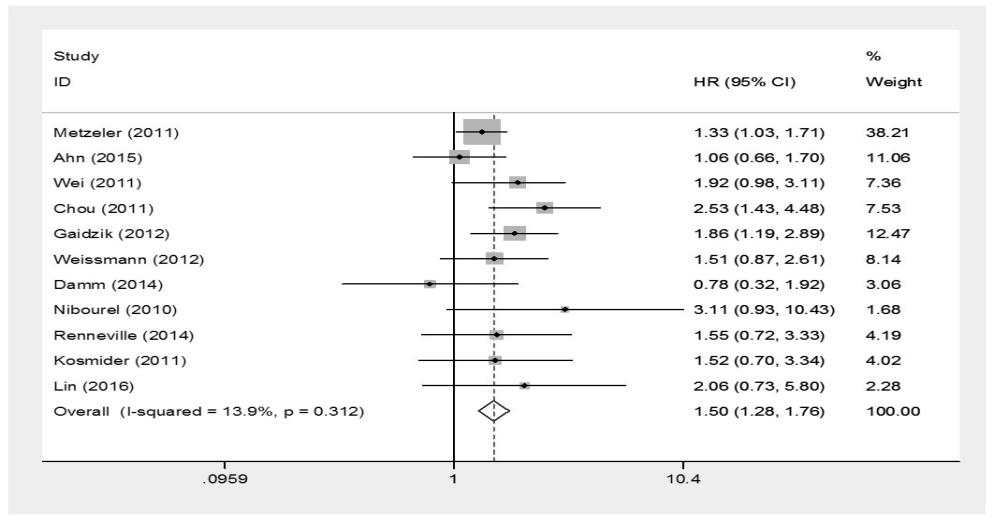


Supplementary Figure S2.


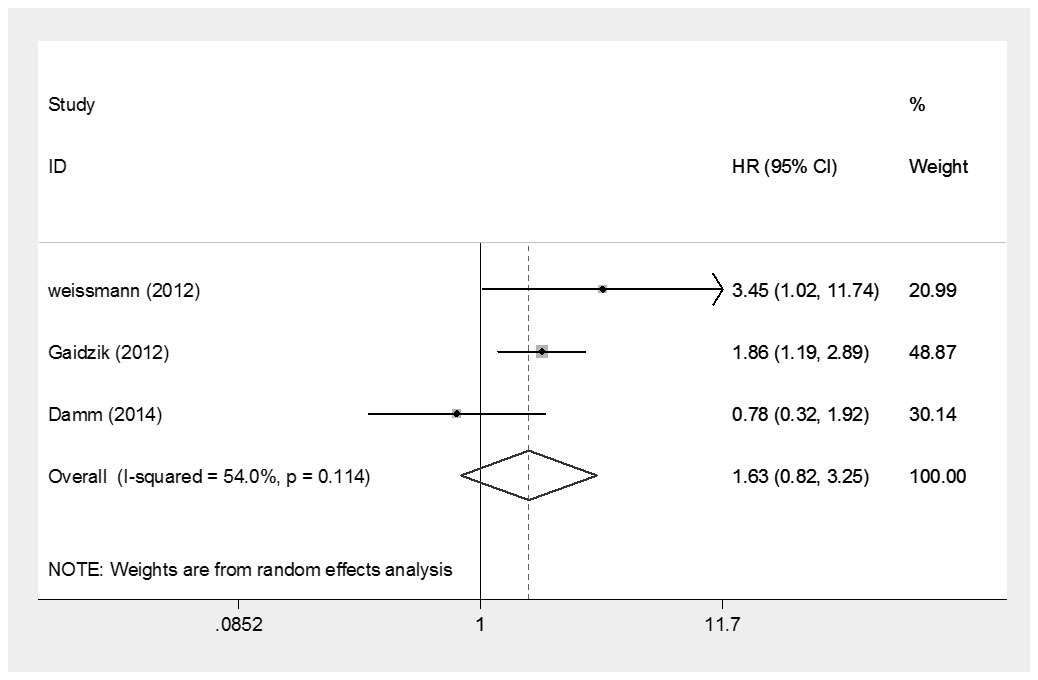


b.


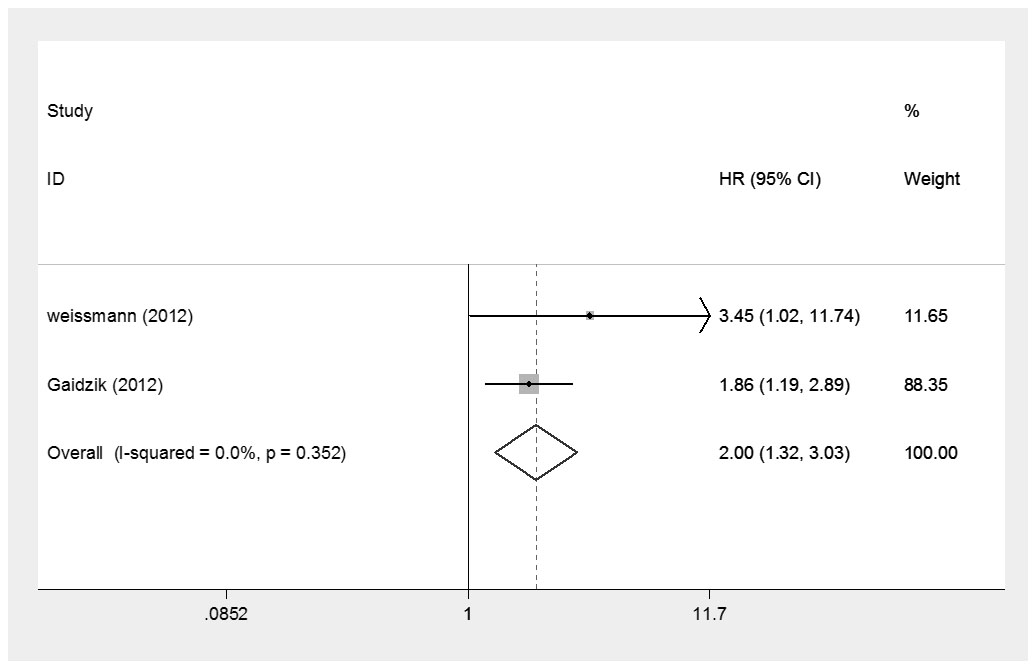


Supplementary Figure S3.

a.


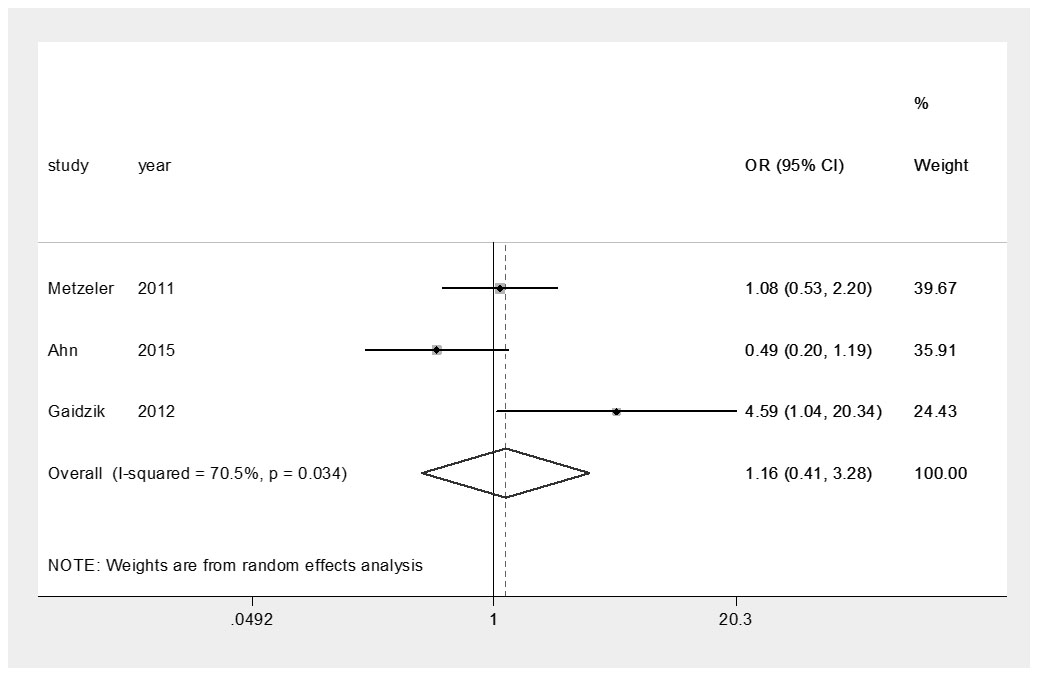


b.


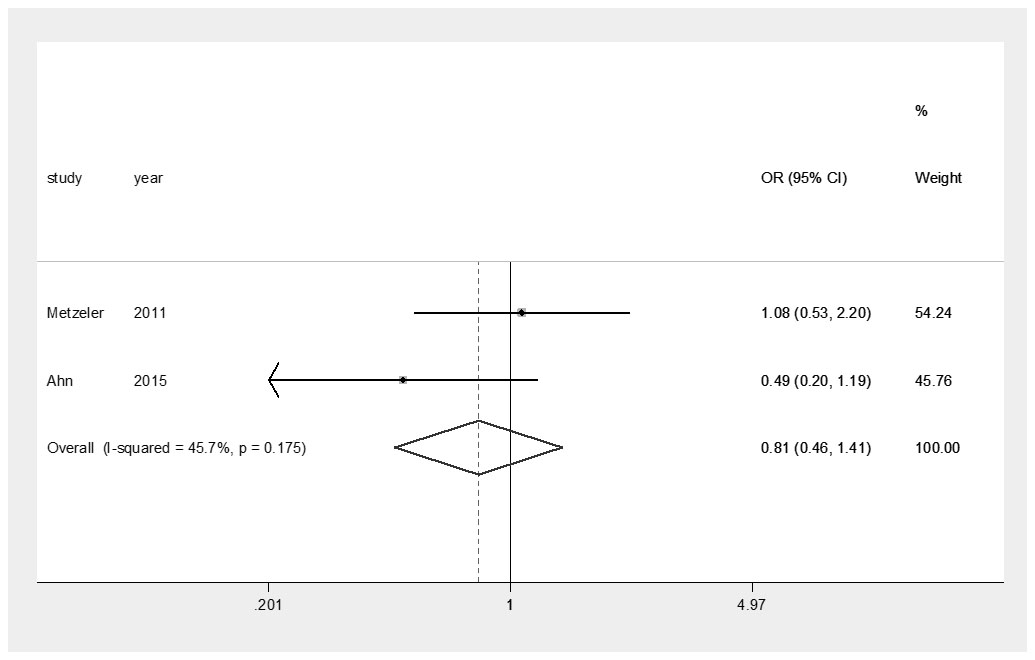


Supplementary Figure S4.

a. b.


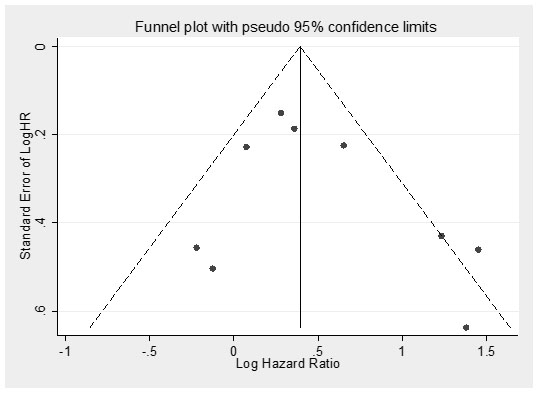

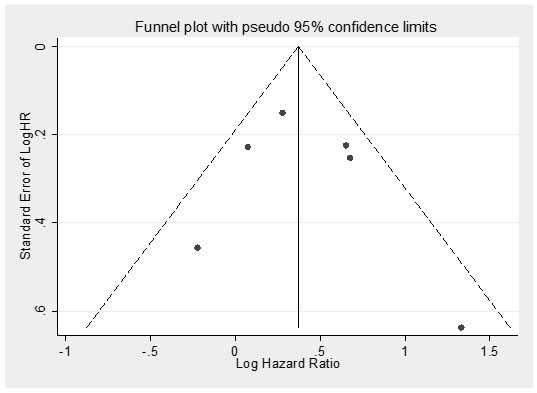


c. d.


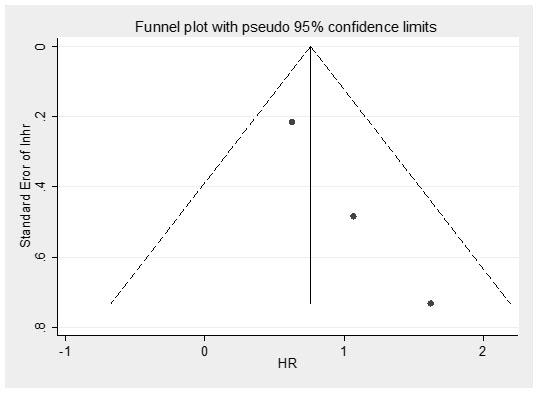

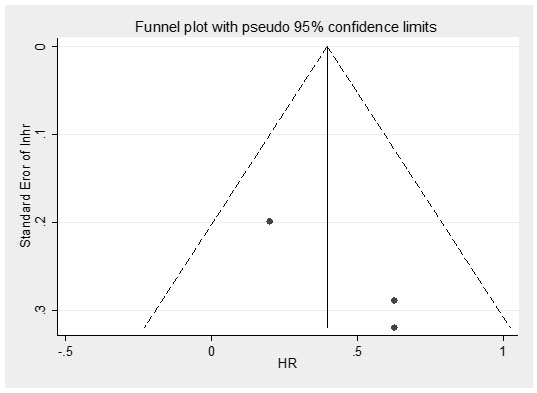

Supplement: Supplementary file 1 — Figure S1: Title of data: Influences of TET2 mutation on prognosis in AML patients. Description of data: forest plots of pooled HRs and 95%CI for OS or EFS in AML patients. Figure S2: Title of data: Influences of TET2 mutation on prognosis in CN-AML patients. Description of data: forest plots of pooled HRs and 95%CI for OS or EFS in CN-AML patients. Figure S3: Title of data: Influences of TET2 mutation on prognosis in patients with ELN intermediate-Irisk cytogenetics. Description of data: forest plots of pooled HRs and 95%CI for OS or EFS of patients with ELN intermediate-Irisk cytogenetics. Figure S4: Title of data: Funnel plot for publication bias test of TET2 mutation in EFS. (DOC 916 kb) [file 12885_2019_5602_MOESM1_ESM.doc]
