# Supplementary material for: The prognostic impact of tet oncogene family member 2 mutations in patients with acute myeloid leukemia: a systematic-review and meta-analysis
Source: BMC Cancer. 2019 Apr 25;19:389. doi: 10.1186/s12885-019-5602-8 (PMC6485112; doi:10.1186/s12885-019-5602-8)
Supplement: Supplementary file 3 — Table S2: Title of data: Patients characteristics from the studies according to TET2 gene status. Table S3: Title of data: The relationship between TET2 mutation and FAB subtypes. Table S4: Title of data: The relationship between TET2 mutation and cytogenetics. Table S5: Title of data: The relationship between TET2 mutation and other genetic aberrations. Table S6: Title of data: The results of Egger test and Begg test to evaluate publication bias. Table S7: Title of data: The results comparison between the fixed effect model and the random effect model. (DOC 356 kb) [file 12885_2019_5602_MOESM3_ESM.doc]

**Supplementary tables**

**Supplementary Table S1.** The specific mutational spots of *TET2* gene collected from 11 studies.

**Supplementary Table S2.** Patients characteristics from the studies according to *TET2* gene status.

**Supplementary Table S3**. The relationship between *TET2* mutation and FAB subtypes.

**Supplementary Table S4**. The relationship between *TET2* mutation and cytogenetics.

**Supplementary Table S5**. The relationship between *TET2* mutation and other genetic aberrations.

**Supplementary Table S6**. The results of Egger test and Begg test to evaluate publication bias.

**Supplementary Table S7**. The results comparison between fixed effect model and random effect model

**Supplementary Table S2.**

| Study | | Median age | | Gender | | | Median WBC count | | Median hemoglobin | | Median platelets | | PB blasts | | BM blasts | |
| --- | --- | --- | --- | --- | --- | --- | --- | --- | --- | --- | --- | --- | --- | --- | --- | --- |
|  |  | Years | *p* value | Male | Female | *p* value | 10^9/L | *p* value | g/dL | *p* value | 10^9/L | *p* value | % | p value | % | *p* value |
| S.Ohgami | TET2mut |  |  |  |  |  |  |  |  |  |  |  |  |  |  |  |
|  | TET2wt |  |  |  |  |  |  |  |  |  |  |  |  |  |  |  |
| Ahn | TET2mut | 62 | <0.001 | 27 | 27 | 0.985 | 46.9 | 0.013 |  |  |  |  | 19.6 | 0.063 | 75 | 0.184 |
|  | TET2wt | 50 |  | 176 | 177 |  | 25.2 |  |  |  |  |  | 6.5 |  | 70 |  |
| Kao | TET2mut | 71 | 0.004 | 10 | 8 | 0.600 | 51.8 | 0.136 | 7.2 | 0.829 | 48 | 0.686 | 64.6 | 0.676 | 77.8 | 0.851 |
|  | TET2wt | 54 |  | 37 | 39 |  | 27.3 |  | 7.4 |  | 46 |  | 62.9 |  | 80.3 |  |
| Cher | TET2mut |  |  |  |  |  |  |  |  |  |  |  |  |  |  |  |
|  | TET2wt |  |  |  |  |  |  |  |  |  |  |  |  |  |  |  |
| Wahab | TET2mut | 70.5 | 0.135 | 5 | 3 | 0.695 |  |  |  |  |  |  |  |  |  |  |
|  | TET2wt | 64 |  | 47 | 38 |  |  |  |  |  |  |  |  |  |  |  |
| Metzeler | TET2mut | 66 | <0.001 | 39 | 56 | 0.06 | 33.5 | 0.04 | 9.3 | 0.59 | 70 | 0.56 | 52 | 0.96 | 67 | 0.86 |
|  | TET2wt | 60 |  | 169 | 154 |  | 24.5 |  | 9.5 |  | 64 |  | 54 |  | 67 |  |
| Chou | TET2mut | 68 | <0.001 | 42 | 22 | 0.136 | 43.16 | <0.001 | 8.1 | 0.196 | 33 | 0.017 |  |  | 26.53 | <0.001 |
|  | TET2wt | 48 |  | 232 | 190 |  | 15.44 |  | 8.0 |  | 45 |  |  |  | 5.80 |  |
| Gaidzik | TET2mut | 51 | 0.08 | 28 | 32 | 0.42 | 20.9 | 0.11 |  |  | 42.5 | 0.11 | 50 | 0.25 | 80 | 0.49 |
|  | TET2wt | 47 |  | 378 | 345 |  | 13.2 |  |  |  | 50 |  | 38 |  | 75 |  |
| Weissmann | TET2mut | 67.5 | <0.001 | 39 | 48 | 0.288 | 29.9 | 0.023 | 9.3 |  | 57.5 | 0.03 |  |  |  |  |
|  | TET2wt | 65.2 |  | 119 | 112 |  | 20.2 |  | 9.2 |  | 58 |  |  |  |  |  |
| Damm | TET2mut | 53 | 0.078 | 5 | 8 | 0.39 | 32.1 | 0.77 | 9.8 | 0.77 | 98.5 | 0.041 |  |  | 80 | 0.99 |
|  | TET2wt | 47 |  | 109 | 93 |  | 26.5 |  | 9 |  | 51 |  |  |  | 80 |  |
| Nibourel | TET2mut | 49.5 | 0.17 | 11 | 9 | 0.33 | 31.6 | 0.14 | 8.9 | 0.45 | 55 | 0.85 |  |  |  |  |
|  | TET2wt | 43 |  | 39 | 52 |  | 13 |  | 8.8 |  | 51 |  |  |  |  |  |
| Kosmider | TET2mut | 71 | <0.001 | 36 | 13 | 0.051 | 20.3 | <0.001 | 9.6 | 0.013 | 39 | 0.003 | 40 | 0.054 | 44 | 0.568 |
|  | TET2wt | 65 |  | 115 | 82 |  | 6.7 |  | 8.9 |  | 64 |  | 29 |  | 50 |  |
| Lin | TET2mut |  |  |  |  |  |  |  |  |  |  |  |  |  |  |  |
|  | TET2wt |  |  |  |  |  |  |  |  |  |  |  |  |  |  |  |
| Renneville | TET2mut |  |  |  |  |  |  |  |  |  |  |  |  |  |  |  |
|  | TET2wt |  |  |  |  |  |  |  |  |  |  |  |  |  |  |  |
| Patel | TET2mut |  |  |  |  |  |  |  |  |  |  |  |  |  |  |  |
|  | TET2wt |  |  |  |  |  |  |  |  |  |  |  |  |  |  |  |
| Tian | TET2mut | 50 | 0.012 | 26 | 34 | 0.109 | 78.6 | 0.686 | 9.6 | 0.016 | 57.6 | 0.749 |  |  | 76 | 0.211 |
|  | TET2wt | 44 |  | 171 | 142 |  | 66.4 |  | 8.6 |  | 58.3 |  |  |  | 72.7 |  |

**Supplementary Table S3.**

| Study | | M0 | M1 | M2 | M3 | M4 | M5 | M6 | M7 | undetermined | *p* value |
| --- | --- | --- | --- | --- | --- | --- | --- | --- | --- | --- | --- |
|  |  |  |  |  |  |  |  |  |  |  |  |
| S.Ohgami | |  |  |  |  |  |  |  |  |  |  |
|  |  |  |  |  |  |  |  |  |  |  |  |
| Ahn | TET2mut | non |  |  |  |  |  |  |  |  |  |
|  | TET2wt |  |  |  |  |  |  |  |  |  |  |
| Kao  * | TET2mut | 0(*p*=0.263) | 5(*p*=0.716) | 8(*p*=0.857) |  | 4(*p*=0.612) | 1(*p*=0.960) | 0(*p*=0.392) | 0(*p*=0.625) |  | 0.873 |
|  | TET2wt | 5 | 18 | 32 |  | 13 | 4 | 3 | 1 |  |  |
| Cher | TET2mut | non |  |  |  |  |  |  |  |  |  |
|  | TET2wt |  |  |  |  |  |  |  |  |  |  |
| Wahab | TET2mut | non |  |  |  |  |  |  |  |  |  |
|  | TET2wt |  |  |  |  |  |  |  |  |  |  |
| Metzeler  * | TET2mut | 0(*p*=0.113) | 14(*p*=0.328) | 22(*p*=0.563) |  | 20(*p*=0.389) | 12(*p*=0.506) | 0(*p*=0.172) |  |  | 0.292 |
|  | TET2wt | 8 | 59 | 64 |  | 54 | 32 | 6 |  |  |  |
|  | TET2wt |  | 12 | 28 |  | 5 | 25 |  |  |  |  |
| Chou  * | TET2mut | 0(*p*=0.208) | 13(*p*=0.632) | 28(*p*=0.125) | 0(*p*=0.005) | 20(*p*=0.245) | 3(*p*=0.905) | 0(*p*=0.187) |  | 0 | 0.065 |
|  | TET2wt | 10 | 95 | 140 | 38 | 101 | 18 | 11 |  | 9 |  |
| Gaidzik | TET2mut | non |  |  |  |  |  |  |  |  |  |
|  | TET2wt |  |  |  |  |  |  |  |  |  |  |
| Weissmann | TET2mut | non |  |  |  |  |  |  |  |  |  |
|  | TET2wt |  |  |  |  |  |  |  |  |  |  |
| Damm  * | TET2mut | 0(*p*=0.522) | 1(*p*=0.311) | 2(*p*=0.456) |  | 8(*p*=0.034) | 2(*p*=0.892) | 0(*p*=0.560) | 0(*p*=0.653) | 0 | 0.52 |
|  | TET2wt | 6 | 37 | 48 |  | 64 | 33 | 5 | 3 | 6 |  |
| Nibourel | TET2mut | non |  |  |  |  |  |  |  |  |  |
|  | TET2wt |  |  |  |  |  |  |  |  |  |  |
| Kosmider | TET2mut | 1 | 6 | 19 | 0 | 9 | 12 | 1 | 1 |  |  |
|  | TET2wt |  |  |  |  |  |  |  |  |  |  |
| Lin | total | 5 | 21 | 37 | 9 | 21 | 6 | 2 | 1 | 10 |  |
|  |  |  |  |  |  |  |  |  |  |  |  |
| Renneville | TET2mut | non |  |  |  |  |  |  |  |  |  |
|  | TET2wt |  |  |  |  |  |  |  |  |  |  |
| Patel | total | 29 | 112 | 115 |  | 63 | 40 | 29 | 3 | 21 |  |
|  |  |  |  |  |  |  |  |  |  |  |  |
| Tian | TET2mut | 0 | 18 | 9 |  | 6 | 8 | 1 |  |  | 0.217 |
|  | TET2wt | 1 | 56 | 65 |  | 29 | 51 | 8 |  |  |  |

**Supplementary Table S4.**

| Study | | Cytogenetics | | | | Normal karyotype | | Complex karyotypes | |
| --- | --- | --- | --- | --- | --- | --- | --- | --- | --- |
|  |  | favorable | intermediate | unfavorable | *p* value | n | *p* value | n | *p* value |
| S.Ohgami | total | 18 | 54 | 21 |  |  |  | 15 |  |
| Ahn | TET2mut |  |  |  |  |  |  |  |  |
|  | TET2wt |  |  |  |  |  |  |  |  |
| Kao  * | TET2mut |  | 13(*p*=0.807) | 0(*p*=0.634) | 0.307 | 56 |  | 1 |  |
|  | TET2wt |  | 60 | 1 |  |  |  |  |  |
| Cher | TET2mut |  |  |  |  |  |  |  |  |
|  | TET2wt |  |  |  |  |  |  |  |  |
| Wahab  * | TET2mut | 0(*p*=0.528) | 4(*p*=0.383) | 4(*p*=0.239) | 0.449 | 1 | 0.166 | 1 | 0.877 |
|  | TET2wt | 4 | 55 | 25 |  | 31 |  | 9 |  |
| Metzeler | TET2mut |  |  |  |  |  |  |  |  |
|  | TET2wt |  |  |  |  |  |  |  |  |
| Chou  * | TET2mut | 5(*p*=0.007) | 45(*p*<0.001) | 11(*p*=0.091) | 0.001 | 39 | 0.006 |  |  |
|  | TET2wt | 91 | 198 | 117 |  | 178 |  |  |  |
| Gaidzik  * | TET2mut | 11(*p*=0.641) | 39(*p*=0.249) | 7(*p*=0.325) | 0.54 |  |  |  |  |
|  | TET2wt | 144 | 398 | 114 |  |  |  |  |  |
| Weissmann | TET2mut |  |  |  |  |  |  |  |  |
|  | TET2wt |  |  |  |  |  |  |  |  |
| Damm | TET2mut |  |  |  |  |  |  |  |  |
|  | TET2wt |  |  |  |  |  |  |  |  |
| Nibourel | TET2mut |  |  |  |  |  |  |  |  |
|  | TET2wt |  |  |  |  |  |  |  |  |
| Kosmider  * | TET2mut |  |  |  |  | 25 | <0.001 | 9 | <0.001 |
|  | TET2wt |  |  |  |  | 44 |  | 87 |  |
| Lin | TET2mut | 1(*p*=0.297) | 8(*p*=0.703) | 3(*p*=0.557) | 0.546 |  |  |  |  |
|  | TET2wt | 21 | 61 | 18 |  |  |  |  |  |
| Renneville | TET2mut |  |  |  |  |  |  |  |  |
|  | TET2wt |  |  |  |  |  |  |  |  |
| Patel | total | 89 | 267 | 122 |  | 244 |  |  |  |
|  |  |  |  |  |  |  |  |  |  |
| Tian | TET2mut |  |  |  |  |  |  |  |  |
|  | TET2wt |  |  |  |  |  |  |  |  |

**Supplementary Table S5.**

| Study | | NPM1 mutation | | FLT3-ITD | | DNMT3A mutation | | IDH mutation | | | CEBPA mutation | | | ASXL1 mutation | |
| --- | --- | --- | --- | --- | --- | --- | --- | --- | --- | --- | --- | --- | --- | --- | --- |
|  |  | n | *p* value | n | *p* value | n | *p* value | n | *p* value | n | | *p* value | n | | *p* value |
| S.Ohgami | TET2mut | 1/6 | 0.970 | 1/6 | 0.970 | 2/6 | 0.195 | 0/6 | 0.231 | 0/6 | | 0.546 | 2/6 | | 0.002 |
|  | TET2wt | 14/87 |  | 14/87 |  | 12/87 |  | 17/87 |  | 5/87 | |  | 3/87 | |  |
| Ahn | TET2mut | 32/54 | 0.017 | 15/54 | 0.929 |  |  |  |  | 11/54 | | 0.910 |  | |  |
|  | TET2wt | 148/353 |  | 96/353 |  |  |  |  |  | 69/350 | |  |  | |  |
| Kao | TET2mut | 0/18 | na | 9/18 | 0.477 | 9/18 | 0.088 | 0/18 | 0.003 |  | |  | 1/18 | | 0.960 |
|  | TET2wt | 0/76 |  | 31/76 |  | 22/76 |  | 27/76 |  |  | |  | 4/76 | |  |
| Cher | TET2mut |  |  |  |  |  |  |  |  |  | |  |  | |  |
|  | TET2wt |  |  |  |  |  |  |  |  |  | |  |  | |  |
| Wahab | TET2mut | 0/8 | 0.489 | 0/8 | 0.255 |  |  |  |  | 0/8 | | 0.437 |  | |  |
|  | TET2wt | 5/85 |  | 12/85 |  |  |  |  |  | 6/85 | |  |  | |  |
| Metzeler | TET2mut | 62/95 | 0.34 | 36/95 | 0.54 |  |  | 4/95 | <0.001 | 20/95 | | 0.07 |  | |  |
|  | TET2wt | 191/323 |  | 110/323 |  |  |  | 122/323 |  | 42/323 | |  |  | |  |
| Chou | TET2mut | 20/64 | 0.047 | 14/64 | 0.847 |  |  | 1/64 | <0.001 |  | |  | 15/64 | | 0.002 |
|  | TET2wt | 82/422 |  | 99/422 |  |  |  | 79/422 |  |  | |  | 38/422 | |  |
| Gaidzik | TET2mut | 18/58 | 0.35 | 17/58 | 0.26 |  |  | 1/60 | <0.001 | 1/33 | | 0.24 |  | |  |
|  | TET2wt | 183/717 |  | 159/701 |  |  |  | 125/723 |  | 43/370 | |  |  | |  |
| Weissmann | TET2mut | 47/85 | 0.145 | 26/85 | 0.388 |  |  |  |  | 4/53 | | 0.098 |  | |  |
|  | TET2wt | 96/209 |  | 56/218 |  |  |  |  |  | 4/158 | |  |  | |  |
| Damm | TET2mut | 11/13 | 0.047 | 4/13 | 0.89 | 10/13 | 0.001 | 0/13 | 0.034 | 1/13 | | 0.43 |  | |  |
|  | TET2wt | 112/198 |  | 65/199 |  | 61/201 |  | 52/200 |  | 30/189 | |  |  | |  |
| Nibourel | TET2mut | 11/20 | 0.03 | 6/20 | 0.19 |  |  |  |  | 2/20 | | 0.98 |  | |  |
|  | TET2wt | 25/91 |  | 14/91 |  |  |  |  |  | 9/91 | |  |  | |  |
| Kosmider | TET2mut | 8/45 | 0.07 |  |  |  |  |  |  |  | |  |  | |  |
|  | TET2wt | 8/103 |  |  |  |  |  |  |  |  | |  |  | |  |
| Lin | TET2mut | 4/12 | 0.046 | 4/12 | 0.429 | 3/12 | 0.166 | 2/12 | 0.953 | 4/12 | | 0.064 | 3/12 | | 0.373 |
|  | TET2wt | 12/100 |  | 23/100 |  | 11/100 |  | 16/100 |  | 13/100 | |  | 15/100 | |  |
| Renneville | TET2mut |  |  |  |  |  |  |  |  |  | |  |  | |  |
|  | TET2wt |  |  |  |  |  |  |  |  |  | |  |  | |  |
| Patel | TET2mut | 10/33 | 0.925 |  |  | 6/32 | 0.417 | 0/33 | 0.008 | 2/32 | | 0.633 | 4/33 | | <0.001 |
|  | TET2wt | 106/359 |  |  |  | 83/349 |  | 56/357 |  | 31/356 | |  | 6/357 | |  |
| Tian | TET2mut | 29/60 | 0.066 | 17/60 | 0.548 |  |  | 4/60 | 0.006 |  | |  | 7/60 | | 0.971 |
|  | TET2wt | 212/313 |  | 101/313 |  |  |  | 69/313 |  |  | |  | 36/313 | |  |

**Supplementary Table S6.**

| Study cohort | Endpoints | Egger’s test | Begg’s test |
| --- | --- | --- | --- |
|  |  | *P* value | *P* value |
| AML | CR rate | 0.483 | 0.452 |
|  | OS | 0.055 | 0.075 |
|  | EFS | 0.290 | 0.466 |
| CN-AML | CR rate | 0.684 | 1.000 |
|  | OS | 0.597 | 1.000 |
|  | EFS | 0.629 | 0.707 |
| IR-AML | OS | 0.465 | 0.640 |
| AML(patients<65 years) | OS | 0.973 | 0.734 |
|  | EFS | 0.665 | 0.734 |
| CN-AML(patients<65 years) | OS | 0.945 | 1.000 |
|  | EFS | 0.727 | 1.000 |
| ELN favorable-risk | CR rate | 0.450 | 1.000 |
|  | OS | 0.677 | 1.000 |
|  | EFS | 0.053 | 0.296 |
| ELN intermediate-risk | CR rate | 0.792 | 1.000 |
|  | EFS | 0.126 | 1.000 |

**Supplementary Table S7.**

| Endpoints | | Fixed effect model | | | | Random effect model | | | |
| --- | --- | --- | --- | --- | --- | --- | --- | --- | --- |
|  |  | HR or OR | *P* value | Heterogeneity, % | *P* value | HR or OR | *P* value | Heterogeneity, % | *P* value |
| AML | CR rate | 0.802[0.583-1.103] | 0.176 | 0 | 0.465 | 0.802[0.583-1.103] | 0.176 | 0 | 0.465 |
|  | OS | 1.417[1.247-1.610] | <0.001 | 34.3 | 0.094 | 1.480[1.241-1.766] | <0.001 | 34.3 | 0.094 |
|  | EFS | 1.480[1.249-1.754] | <0.001 | 56.4 | 0.019 | 1.594[1.187-2.141] | 0.002 | 56.4 | 0.019 |
| CN-AML | CR rate | 0.803[0.562-1.147] | 0.228 | 39.0 | 0.161 | 0.840[0.502-1.404] | 0.505 | 39.0 | 0.161 |
|  | OS | 1.425[1.221-1.664] | <0.001 | 0 | 0.633 | 1.425[1.221-1.664] | <0.001 | 0 | 0.633 |
|  | EFS | 1.450[1.199-1.754] | <0.001 | 45.1 | 0.105 | 1.483[1.115-1.974] | 0.007 | 45.1 | 0.105 |
| IR-AML | OS | 1.589[1.363-1.854] | <0.001 | 46.1 | 0.046 | 1.662[1.312-2.105] | <0.001 | 46.1 | 0.046 |
| AML  (patients<65 years) | OS | 1.310[0.999-1.718] | 0.051 | 24.6 | 0.264 | 1.293[0.852-1.961] | 0.227 | 24.6 | 0.264 |
|  | EFS | 1.593[1.185-2.143] | 0.002 | 56.1 | 0.077 | 1.724[1.007-2.954] | 0.047 | 56.1 | 0.077 |
| CN-AML  (patients<65 years) | OS | 1.690[1.158-2.467] | 0.006 | 54.0 | 0.114 | 1.630[0.817-3.250] | 0.166 | 54.0 | 0.114 |
|  | EFS | 1.741[1.180-2.569] | 0.005 | 47.6 | 0.148 | 1.655[0.918-2.985] | 0.094 | 47.6 | 0.148 |
| ELN favorable-risk | CR rate | 0.460[0.252-0.840] | 0.011 | 0 | 0.405 | 0.455[0.245-0.844] | 0.013 | 0 | 0.405 |
|  | OS | 2.034[1.440-2.872] | <0.001 | 0 | 0.797 | 2.034[1.440-2.872] | <0.001 | 0 | 0.797 |
|  | EFS | 2.140[1.476-3.101] | <0.001 | 8.8 | 0.334 | 2.211[1.445-3.385] | <0.001 | 8.8 | 0.334 |
| ELN intermediate-risk | CR rate | 1.120[0.679-1.846] | 0.657 | 70.5 | 0.034 | 1.158[0.408-3.280] | 0.783 | 70.5 | 0.034 |
|  | EFS | 1.487[1.117-1.978] | 0.007 | 6.3 | 0.334 | 1.496[1.111-2.019] | 0.008 | 6.3 | 0.334 |
